# Perianal squamous cell carcinoma in the absence of HPV or immunosuppression: A rare case report

**DOI:** 10.1016/j.jdcr.2026.07.016

**Published:** 2026-07-18

**Authors:** Vincent Doan, Claudia Lee, Angela J. Jiang, Alex G. Ortega-Loayza

**Affiliations:** a Lincoln Memorial University-DeBusk College of Osteopathic Medicine, Knoxville, Tennessee; b Department of Dermatology, Oregon Health and Science University, Portland, Oregon

**Keywords:** biopsy challenges, chronic inflammation, dermatopathology, inverse psoriasis, perianal squamous cell carcinoma

## Introduction

Cutaneous malignancies of the anal margin are rare, with squamous cell carcinoma (SCC) being the most common.^1^^,^^2^ Perianal SCCs present as a slow-growing perianal mass, often accompanied by pain and bleeding in a majority of patients.^2^ Due to its rarity and nonspecific clinical presentation, 70% to 80% of perianal SCCs are frequently misdiagnosed as other benign anorectal conditions, such as hemorrhoids.^2^ This leads to significant diagnostic delays and subsequent therapeutic challenges that negatively impact morbidity and mortality.^2^, ^3^, ^4^ Recognizing high-risk factors, such as immunosuppression and human papillomavirus (HPV), which together account for 86% to 97% of perianal SCCs, can lead to earlier diagnosis and improved outcomes.^5^ Other frequently reported risk factors include smoking and a history of sexually transmitted diseases.^5^^,^^6^ However, in the absence of these high-risk factors, it is important to consider other potentially underrecognized causes of perianal SCC, such as chronic inflammatory dermatoses.^7^, ^8^, ^9^

Perianal SCC secondary to chronic inflammation has been linked to several skin conditions, most notably hidradenitis suppurativa (HS), lichen sclerosus, and cutaneous Crohn’s disease.^7^, ^8^, ^9^, ^10^ Although SCC development has been well-documented in other psoriatic subtypes,^11^, ^12^, ^13^ no cases of perianal SCC arising in the setting of inverse psoriasis (IP) have been reported to date. Because inflammation-driven SCCs portend a worse prognosis, coupled with the inherent challenges in diagnosing perianal malignancies, it is important to recognize this rare, yet potentially fatal complication associated with untreated IP.^13^^,^^14^ Here, we present a rare case of advanced perianal SCC arising in chronically undertreated IP. This case highlights the importance of early and sustained management of IP, maintaining suspicion for malignancy in chronically inflamed perianal skin, and recognizing biopsy-related diagnostic challenges that may delay diagnosis of perianal SCC.^8^

## Case report

A 70-year-old male presented to our clinic with a large ulcerative vegetative perianal mass characterized by bleeding and pain that had been progressing over 3 years. His prior medical history was only notable for a 5 year history of IPinfrequently treated with topical steroids. For the mass, the patient had been previously evaluated by several specialties at outside facilities, including dermatology, infectious diseases, general surgery, and gastroenterology. Prior workup included 2 skin biopsieswhich demonstrated dense mixed inflammation and pseudocarcinomatous hyperplasia (PEH), raising suspicion for an underlying infection. However, an extensive infectious workup including special stains, serology, and wound cultures ruled out atypical fungal infections, hepatitis B and C, chlamydia, herpes simplex virus, syphilis, HPV, and human immunodeficiency virus (HIV). A superficial MRSA infection was treated with antibiotics. Colonoscopy was unremarkable. Previous treatments, including topical and oral antibiotics, triamcinolone, topical lidocaine, and benzoyl peroxide, had failed to provide adequate improvement.

**Fig 1:**
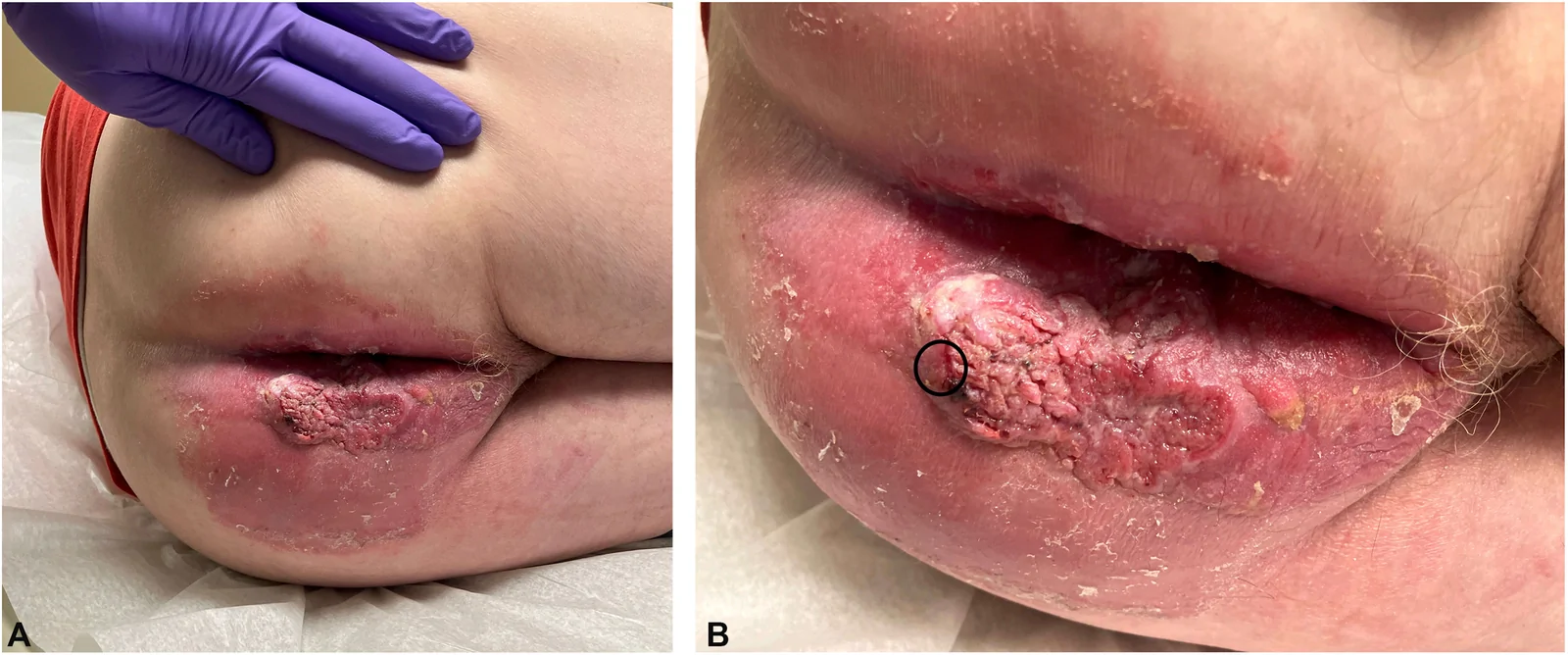
**A,** Located on the left medial buttock and extending into the intergluteal cleft is a 5.5 cm × 4.5 cm ulcerated, friable, cauliflower-like mass superimposed on a well-demarcated erythematous scaly plaque. **B,** A 6-mm punch biopsy was taken from the non-ulcerated border (*black circle*) of the papillomatous perianal mass.

**Fig 2:**
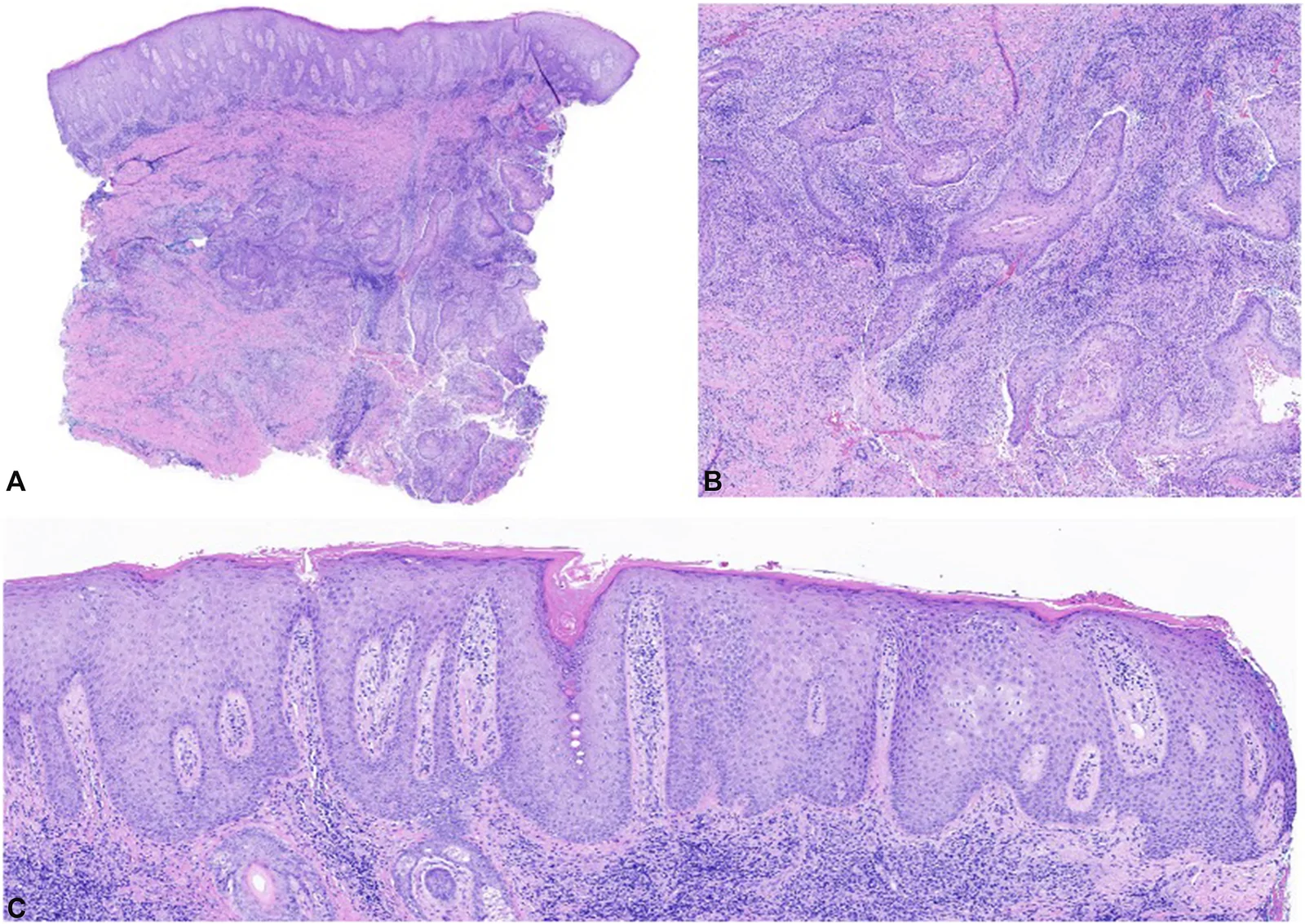
**A,** Punch biopsy demonstrating a dermal proliferation of irregular aggregates of atypical keratinocytes with overlying psoriasiform epidermal hyperplasia (2×). **B,** There are irregular aggregates of atypical keratinocytes admixed with dense mixed inflammation (40×). **C,** There is regular psoriasiform acanthosis with foci of neutrophils within the stratum corneum. There is underlying lichenoid inflammation (80×).

Our initial exam revealed a large, well-demarcated erythematous plaque on the left buttocks with a superimposed 5.5 cm × 4.5 cm ulcerated, friable, cauliflower-like mass that extended into the intergluteal cleft (Fig 1, *A*). Additionally, 2 erythematous, punched-out ulcers with vesicles were present on the glans penis. A 6-mm punch biopsy was taken from the papillomatous border adjacent to the centrally ulcerating mass as seen in (Fig 1, *B*), which revealed extensive atypical squamous proliferation within the dermis, consistent with squamous cell carcinoma (Fig 2, *A*-*C*). Overlying epidermal changes, including regular psoriasiform acanthosis with neutrophils in the stratum corneum, supported the underlying diagnosis of IP (Fig 2, C). Following further diagnostic evaluation and histopathologic confirmation of SCC, the patient was found to have involvement of both the internal and external anal sphincters and was subsequently treated with surgical resection in combination with chemoradiation.

## Discussion

This case highlights the diagnostic and management complexities of perianal SCC, especially in a patient with IP without typical risk factors, where profound inflammation further complicates and obscures the diagnosis. It is hypothesized that chronically inflamed skin fosters the concept of an “immunocompromised cutaneous district” where local immune control becomes dysregulated, leading to malignant transformation.^15^ Patients with perianal SCC often experience prolonged misdiagnosis and ineffective treatments that further complicate disease progression.^2^^,^^8^ Studies have shown that diagnostic delays can lead to metastasis.^7^^,^^8^ Factors that lead to delayed diagnosis include non-specific symptoms, absence of typical risk factors, biopsy sampling errors, and lack of routine screening.^6^^,^^8^, ^9^, ^10^ Anogenital SCCs demonstrate increased susceptibility to superficial infections, particularly in the context of local immune dysregulation and immunosuppression. This susceptibility can both potentiate inflammation and complicate histopathological interpretation. Reflecting these challenges, a 2024 study reported that 71% of patients required multiple biopsies to confirm a diagnosis of SCC secondary to HS.^10^

Current literature on SCC arising in the setting of psoriasis remains limited. Nevertheless, meta-analyses and observational studies consistently demonstrate that patients with psoriasis have an increased risk of non-melanoma skin cancers (NMSCs), including both SCC and basal cell carcinoma.^11^^,^^12^^,^^16^ This elevated risk has been largely attributed to treatment-related exposures, particularly immunosuppressive therapies and prior phototherapy. Among these factors, psoralen ultraviolet A (PUVA) therapy has been most associated with a highly elevated risk of SCC, with studies reporting markedly increased standardized incidence ratios.^11^^,^^13^ Additional risk has been linked to systemic agents such as cyclosporine and, to a lesser extent, methotrexate. Current guidelines, including those from the American Academy of Dermatology (AAD), highlight a clear association between PUVA exposure and SCC, while cyclosporine appears to increase malignancy risk independent of the underlying disease.^14^

Beyond treatment-related factors, individuals with psoriasis have a higher prevalence of established carcinogenic risk factors, including smoking, excessive alcohol consumption, and obesity, which may further contribute to the observed increase in SCC incidence.^13^ In the absence of these exposures, chronic immune dysregulation and sustained cutaneous inflammation inherent to psoriasis may create a permissive microenvironment for malignant transformation, positioning chronic inflammation as a potential primary driver of SCC in select cases lacking traditional risk factors.^11^^,^^13^

In the case discussed, the patient underwent months of unsuccessful wound care and non-diagnostic biopsies before receiving the correct diagnosis. Diagnostic accuracy in ulcerated lesions is challenging, as biopsy site selection critically influences yield.^2^^,^^17^ Biopsies are conventionally taken from the periphery of an ulceration to avoid the non-specific inflammatory changes seen beneath an ulcer. However, superficial or peripheral sampling can miss invasive disease, and in this case, given the prior non-diagnostic results, a repeat biopsy was targeted toward the center of the lesion with deep sampling to obtain sufficient tissue beyond superficial dermis. This approach highlights the importance of considering multiple biopsies from different sites when initial sampling is unrevealing.^2^^,^^17^ To ensure prompt, accurate detection of perianal SCC, a structured, multidisciplinary strategy is essential. Clinicians must first consider benign anorectal conditions such as hemorrhoids, anal fissures, and perianal abscesses, while also ruling out other malignancies like anal adenocarcinoma, malignant melanoma, and lymphoma.^2^^,^^18^ Immunohistochemical markers can aid in differentiating these entities.^18^ In addition, infectious and inflammatory causes, including Crohn disease, syphilis, tuberculosis, and granulomatous processes, should be carefully considered.^2^^,^^7^^,^^9^

## Conclusion

Chronic inflammatory dermatoses, such as inverse psoriasis, of the anogenital region may obscure or contribute to the development of SCC. Therefore, clinicians should maintain suspicion for squamous cell carcinoma even when classic risk factors are absent. In lesions that fail to respond to conventional therapies or yield non-diagnostic biopsies, careful site selection or repeat targeted sampling should be pursued to avoid diagnostic delays.

## Conflicts of interest

None disclosed.
